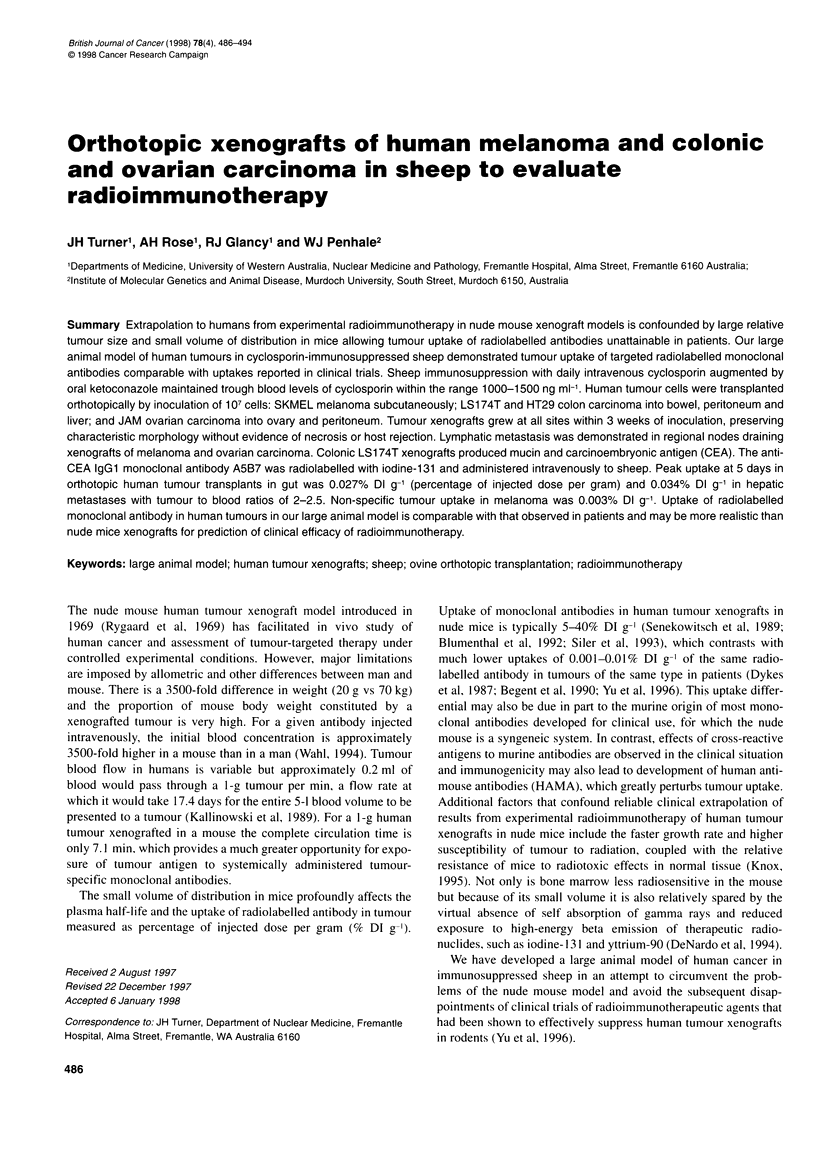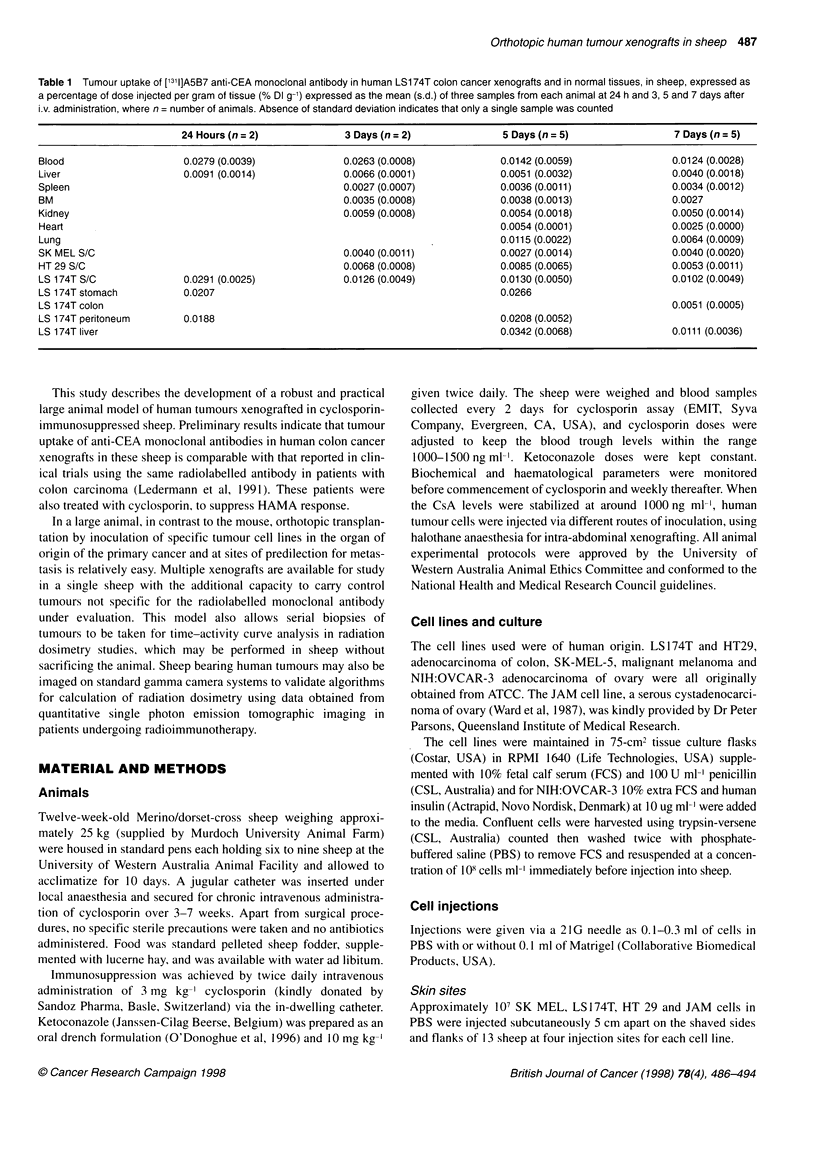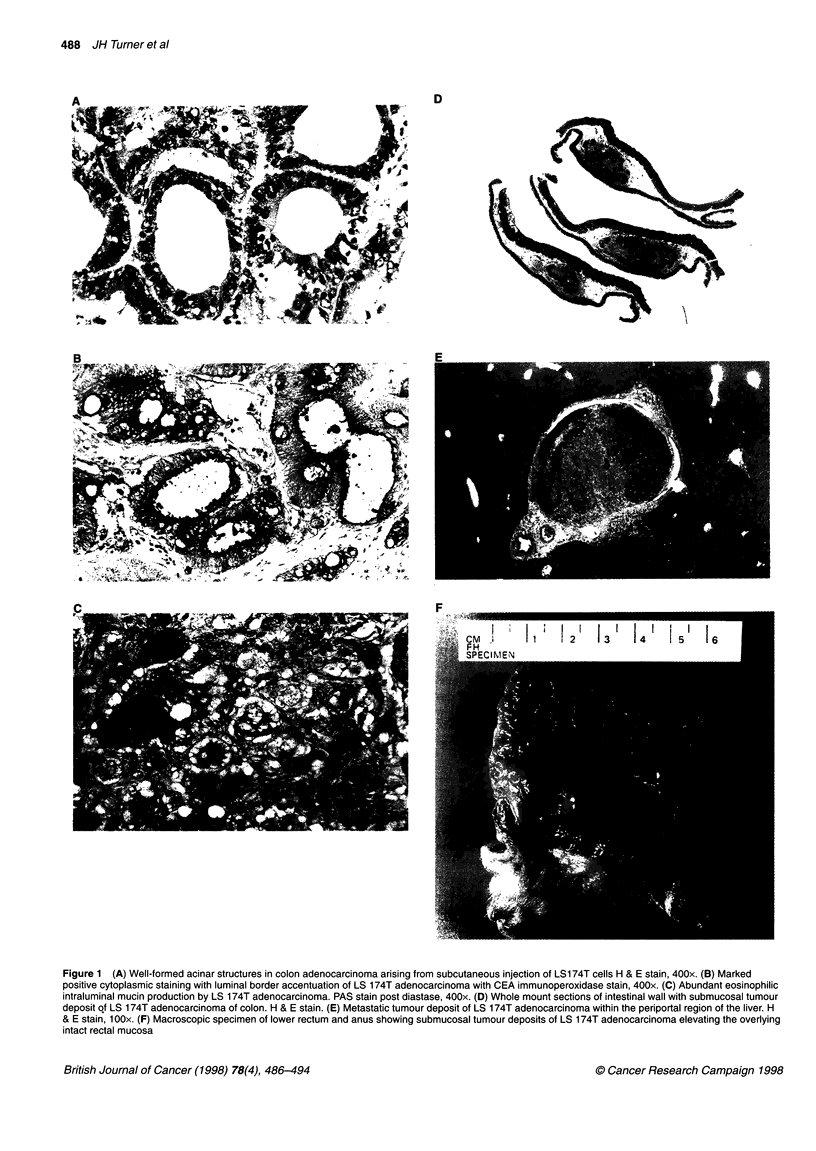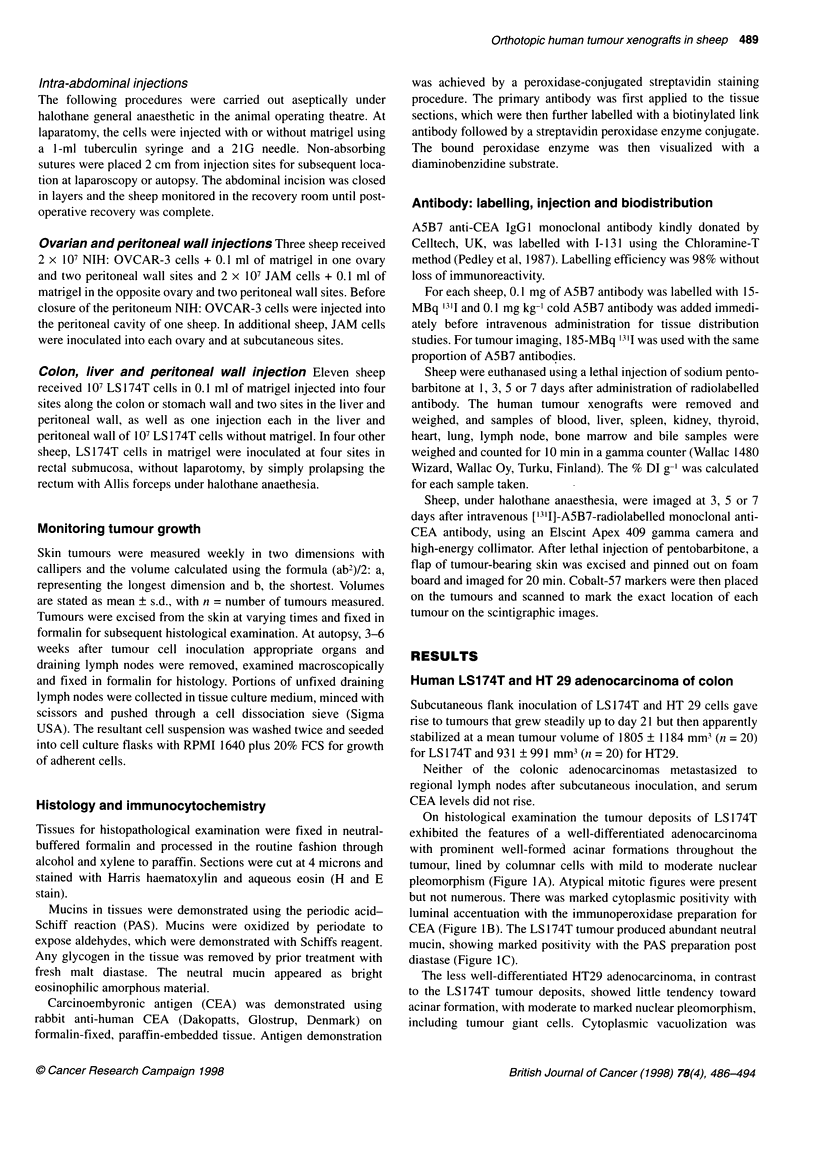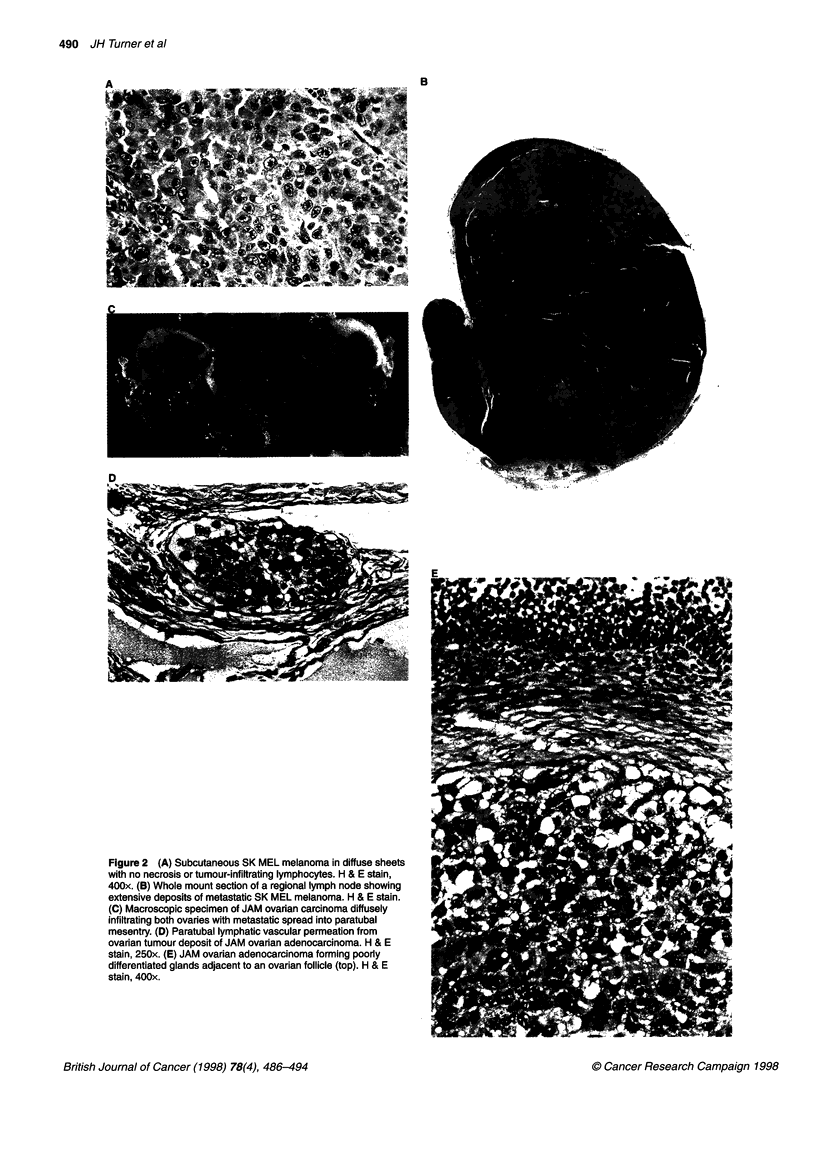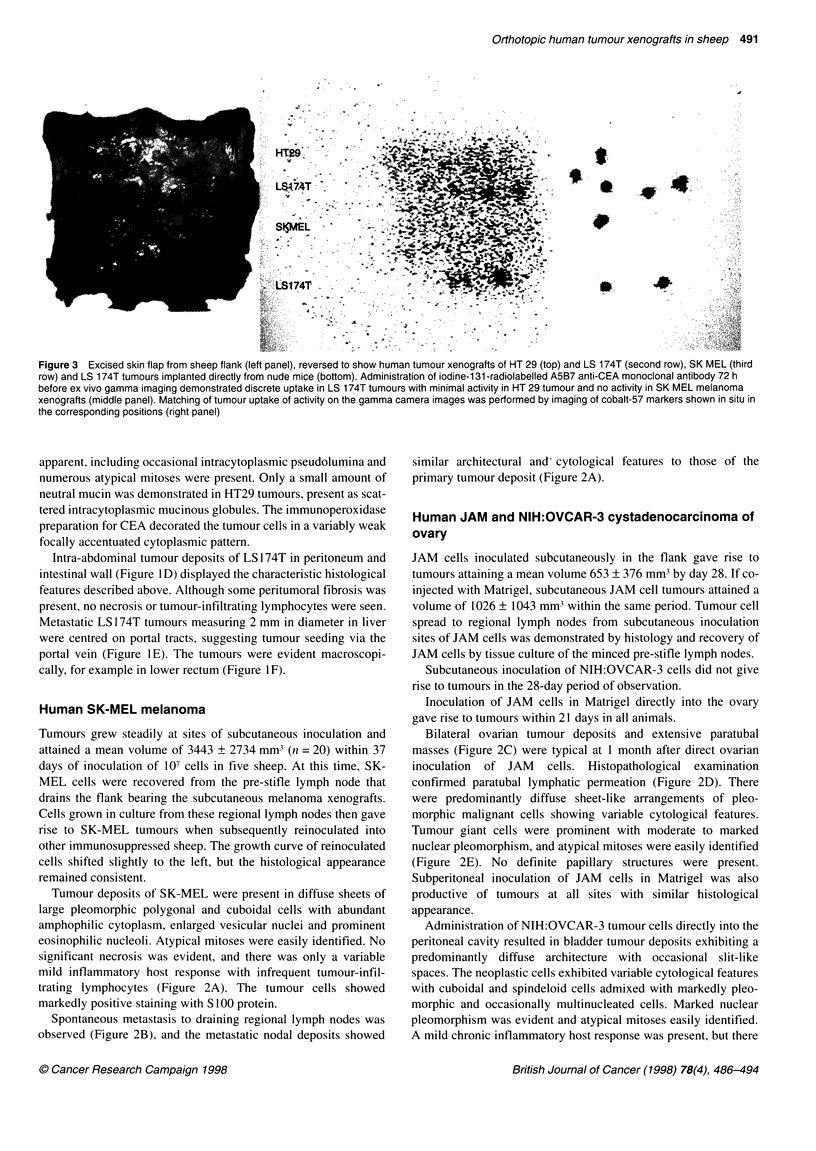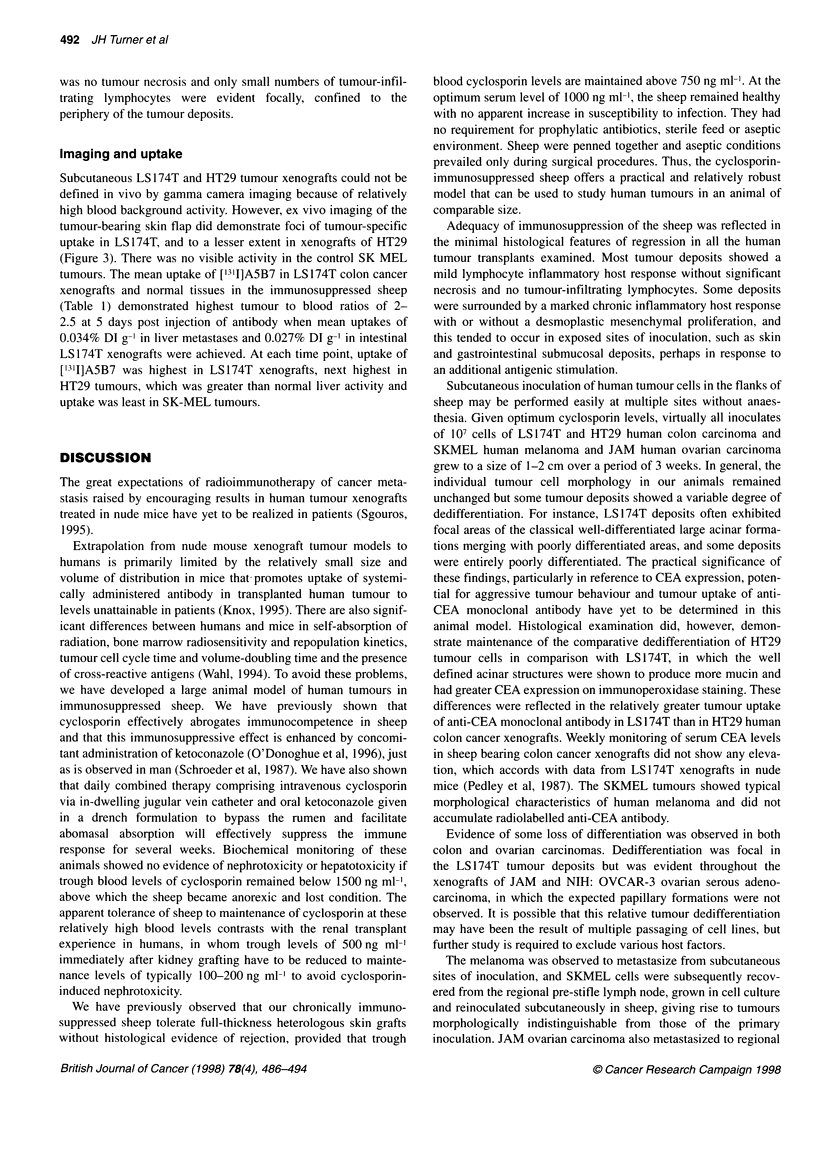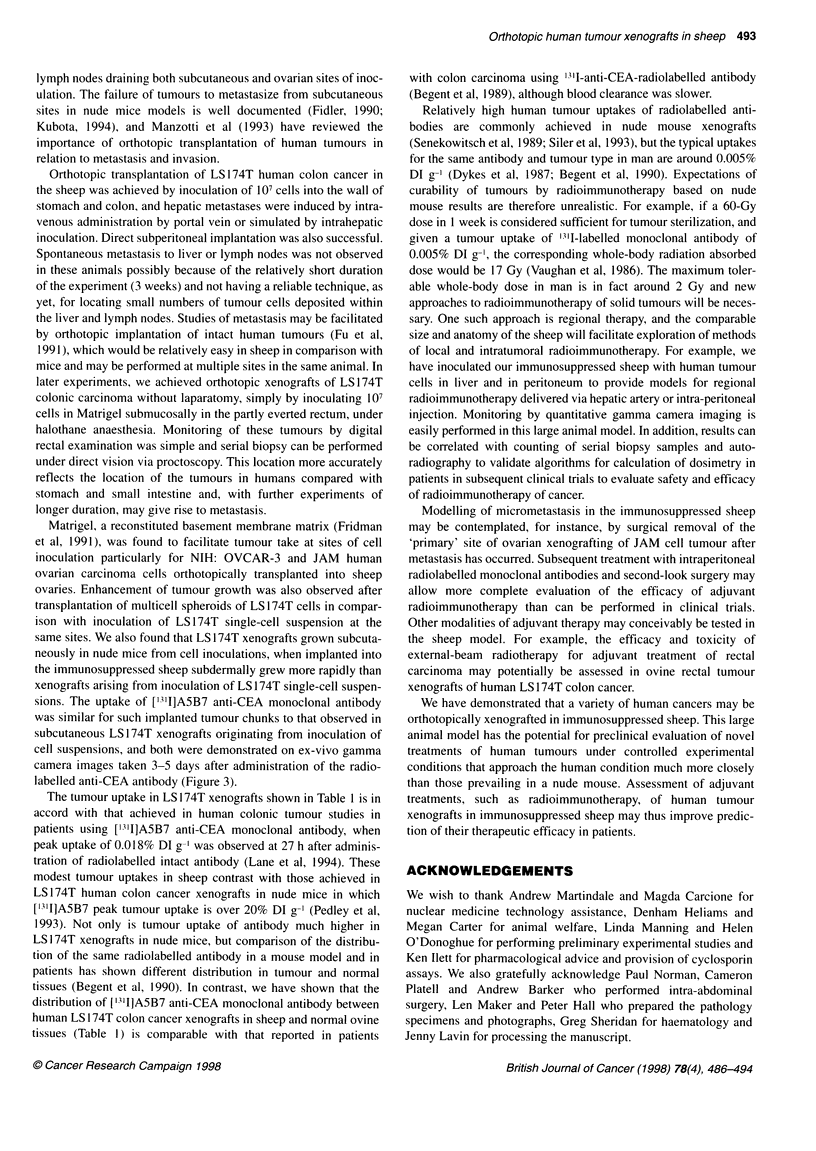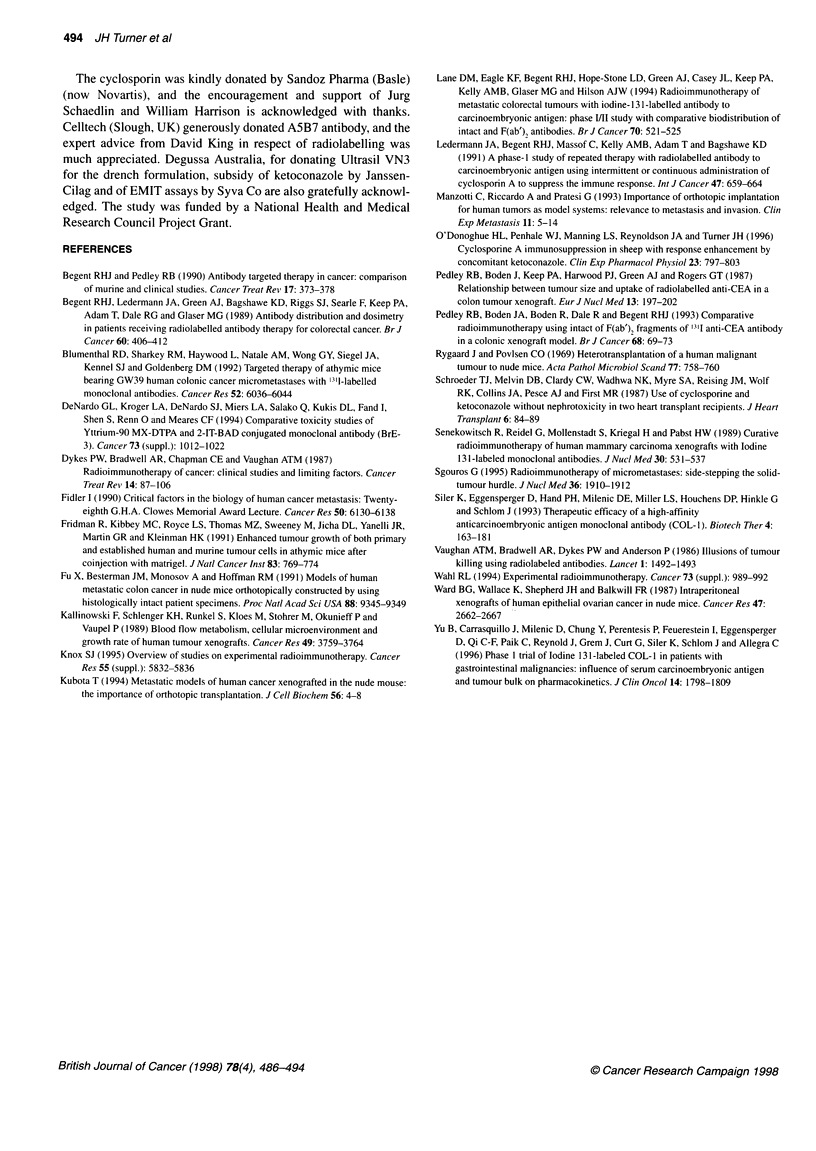# Orthotopic xenografts of human melanoma and colonic and ovarian carcinoma in sheep to evaluate radioimmunotherapy.

**DOI:** 10.1038/bjc.1998.520

**Published:** 1998-08

**Authors:** J. H. Turner, A. H. Rose, R. J. Glancy, W. J. Penhale

**Affiliations:** Department of Medicine, University of Western Australia, Fremantle Hospital, Australia.

## Abstract

**Images:**


British Journal of Cancer (1998) 78(4), 486–494
© 1998 Cancer Research Campaign

### Orthotopic xenografts of human melanoma and colonic and ovarian carcinoma in sheep to evaluate radioimmunotherapy

**JH Turner[1], AH Rose[1], RJ Glancy[1] and WJ Penhale[2]**

[1]Departments of Medicine, University of Western Australia, Nuclear Medicine and Pathology, Fremantle Hospital, Alma Street, Fremantle 6160 Australia; [2]Institute of Molecular Genetics and Animal Disease, Murdoch University, South Street, Murdoch 6150, Australia

**Summary** Extrapolation to humans from experimental radioimmunotherapy in nude mouse xenograft models is confounded by large relative tumour size and small volume of distribution in mice allowing tumour uptake of radiolabelled antibodies unattainable in patients. Our large animal model of human tumours in cyclosporin-immunosuppressed sheep demonstrated tumour uptake of targeted radiolabelled monoclonal antibodies comparable with uptakes reported in clinical trials. Sheep immunosuppression with daily intravenous cyclosporin augmented by oral ketoconazole maintained trough blood levels of cyclosporin within the range 1000–1500 ng $ml^{-1}$. Human tumour cells were transplanted orthotopically by inoculation of $10^7$ cells: SKMEL melanoma subcutaneously; LS174T and HT29 colon carcinoma into bowel, peritoneum and liver; and JAM ovarian carcinoma into ovary and peritoneum. Tumour xenografts grew at all sites within 3 weeks of inoculation, preserving characteristic morphology without evidence of necrosis or host rejection. Lymphatic metastasis was demonstrated in regional nodes draining xenografts of melanoma and ovarian carcinoma. Colonic LS174T xenografts produced mucin and carcinoembryonic antigen (CEA). The anti-CEA IgG1 monoclonal antibody A5B7 was radiolabelled with iodine-131 and administered intravenously to sheep. Peak uptake at 5 days in orthotopic human tumour transplants in gut was 0.027% DI $g^{-1}$ (percentage of injected dose per gram) and 0.034% DI $g^{-1}$ in hepatic metastases with tumour to blood ratios of 2–2.5. Non-specific tumour uptake in melanoma was 0.003% DI $g^{-1}$. Uptake of radiolabelled monoclonal antibody in human tumours in our large animal model is comparable with that observed in patients and may be more realistic than nude mice xenografts for prediction of clinical efficacy of radioimmunotherapy.

**Keywords:** large animal model; human tumour xenografts; sheep; ovine orthotopic transplantation; radioimmunotherapy

The nude mouse human tumour xenograft model introduced in 1969 (Rygaard et al, 1969) has facilitated in vivo study of human cancer and assessment of tumour-targeted therapy under controlled experimental conditions. However, major limitations are imposed by allometric and other differences between man and mouse. There is a 3500-fold difference in weight (20 g vs 70 kg) and the proportion of mouse body weight constituted by a xenografted tumour is very high. For a given antibody injected intravenously, the initial blood concentration is approximately 3500-fold higher in a mouse than in a man (Wahl, 1994). Tumour blood flow in humans is variable but approximately 0.2 ml of blood would pass through a 1-g tumour per min, a flow rate at which it would take 17.4 days for the entire 5-l blood volume to be presented to a tumour (Kallinowski et al, 1989). For a 1-g human tumour xenografted in a mouse the complete circulation time is only 7.1 min, which provides a much greater opportunity for exposure of tumour antigen to systemically administered tumour-specific monoclonal antibodies.

The small volume of distribution in mice profoundly affects the plasma half-life and the uptake of radiolabelled antibody in tumour measured as percentage of injected dose per gram (% DI $g^{-1}$).

*Received 2 August 1997*
*Revised 22 December 1997*
*Accepted 6 January 1998*

*Correspondence to:* JH Turner, Department of Nuclear Medicine, Fremantle Hospital, Alma Street, Fremantle, WA Australia 6160

Uptake of monoclonal antibodies in human tumour xenografts in nude mice is typically 5–40% DI $g^{-1}$ (Senekowitsch et al, 1989; Blumenthal et al, 1992; Siler et al, 1993), which contrasts with much lower uptakes of 0.001–0.01% DI $g^{-1}$ of the same radiolabelled antibody in tumours of the same type in patients (Dykes et al, 1987; Begent et al, 1990; Yu et al, 1996). This uptake differential may also be due in part to the murine origin of most monoclonal antibodies developed for clinical use, for which the nude mouse is a syngeneic system. In contrast, effects of cross-reactive antigens to murine antibodies are observed in the clinical situation and immunogenicity may also lead to development of human anti-mouse antibodies (HAMA), which greatly perturbs tumour uptake. Additional factors that confound reliable clinical extrapolation of results from experimental radioimmunotherapy of human tumour xenografts in nude mice include the faster growth rate and higher susceptibility of tumour to radiation, coupled with the relative resistance of mice to radiotoxic effects in normal tissue (Knox, 1995). Not only is bone marrow less radiosensitive in the mouse but because of its small volume it is also relatively spared by the virtual absence of self absorption of gamma rays and reduced exposure to high-energy beta emission of therapeutic radionuclides, such as iodine-131 and yttrium-90 (DeNardo et al, 1994).

We have developed a large animal model of human cancer in immunosuppressed sheep in an attempt to circumvent the problems of the nude mouse model and avoid the subsequent disappointments of clinical trials of radioimmunotherapeutic agents that had been shown to effectively suppress human tumour xenografts in rodents (Yu et al, 1996).

**Table 1** Tumour uptake of [$^{131}$I]A5B7 anti-CEA monoclonal antibody in human LS174T colon cancer xenografts and in normal tissues, in sheep, expressed as a percentage of dose injected per gram of tissue (% DI $g^{-1}$) expressed as the mean (s.d.) of three samples from each animal at 24 h and 3, 5 and 7 days after i.v. administration, where *n* = number of animals. Absence of standard deviation indicates that only a single sample was counted

| | 24 Hours (*n* = 2) | 3 Days (*n* = 2) | 5 Days (*n* = 5) | 7 Days (*n* = 5) |
|---|---|---|---|---|
| Blood | 0.0279 (0.0039) | 0.0263 (0.0008) | 0.0142 (0.0059) | 0.0124 (0.0028) |
| Liver | 0.0091 (0.0014) | 0.0066 (0.0001) | 0.0051 (0.0032) | 0.0040 (0.0018) |
| Spleen | | 0.0027 (0.0007) | 0.0036 (0.0011) | 0.0034 (0.0012) |
| BM | | 0.0035 (0.0008) | 0.0038 (0.0013) | 0.0027 |
| Kidney | | 0.0059 (0.0008) | 0.0054 (0.0018) | 0.0050 (0.0014) |
| Heart | | | 0.0054 (0.0001) | 0.0025 (0.0000) |
| Lung | | | 0.0115 (0.0022) | 0.0064 (0.0009) |
| SK MEL S/C | | 0.0040 (0.0011) | 0.0027 (0.0014) | 0.0040 (0.0020) |
| HT 29 S/C | | 0.0068 (0.0008) | 0.0085 (0.0065) | 0.0053 (0.0011) |
| LS 174T S/C | 0.0291 (0.0025) | 0.0126 (0.0049) | 0.0130 (0.0050) | 0.0102 (0.0049) |
| LS 174T stomach | 0.0207 | | 0.0266 | |
| LS 174T colon | | | | 0.0051 (0.0005) |
| LS 174T peritoneum | 0.0188 | | 0.0208 (0.0052) | |
| LS 174T liver | | | 0.0342 (0.0068) | 0.0111 (0.0036) |

This study describes the development of a robust and practical large animal model of human tumours xenografted in cyclosporin-immunosuppressed sheep. Preliminary results indicate that tumour uptake of anti-CEA monoclonal antibodies in human colon cancer xenografts in these sheep is comparable with that reported in clinical trials using the same radiolabelled antibody in patients with colon carcinoma (Ledermann et al, 1991). These patients were also treated with cyclosporin, to suppress HAMA response.

In a large animal, in contrast to the mouse, orthotopic transplantation by inoculation of specific tumour cell lines in the organ of origin of the primary cancer and at sites of predilection for metastasis is relatively easy. Multiple xenografts are available for study in a single sheep with the additional capacity to carry control tumours not specific for the radiolabelled monoclonal antibody under evaluation. This model also allows serial biopsies of tumours to be taken for time–activity curve analysis in radiation dosimetry studies, which may be performed in sheep without sacrificing the animal. Sheep bearing human tumours may also be imaged on standard gamma camera systems to validate algorithms for calculation of radiation dosimetry using data obtained from quantitative single photon emission tomographic imaging in patients undergoing radioimmunotherapy.

### MATERIAL AND METHODS

### Animals

Twelve-week-old Merino/dorset-cross sheep weighing approximately 25 kg (supplied by Murdoch University Animal Farm) were housed in standard pens each holding six to nine sheep at the University of Western Australia Animal Facility and allowed to acclimatize for 10 days. A jugular catheter was inserted under local anaesthesia and secured for chronic intravenous administration of cyclosporin over 3–7 weeks. Apart from surgical procedures, no specific sterile precautions were taken and no antibiotics administered. Food was standard pelleted sheep fodder, supplemented with lucerne hay, and was available with water ad libitum.

Immunosuppression was achieved by twice daily intravenous administration of 3 mg $kg^{-1}$ cyclosporin (kindly donated by Sandoz Pharma, Basle, Switzerland) via the in-dwelling catheter. Ketoconazole (Janssen-Cilag Beerse, Belgium) was prepared as an oral drench formulation (O'Donoghue et al, 1996) and 10 mg $kg^{-1}$ given twice daily. The sheep were weighed and blood samples collected every 2 days for cyclosporin assay (EMIT, Syva Company, Evergreen, CA, USA), and cyclosporin doses were adjusted to keep the blood trough levels within the range 1000–1500 ng $ml^{-1}$. Ketoconazole doses were kept constant. Biochemical and haematological parameters were monitored before commencement of cyclosporin and weekly thereafter. When the CsA levels were stabilized at around 1000 ng $ml^{-1}$, human tumour cells were injected via different routes of inoculation, using halothane anaesthesia for intra-abdominal xenografting. All animal experimental protocols were approved by the University of Western Australia Animal Ethics Committee and conformed to the National Health and Medical Research Council guidelines.

### Cell lines and culture

The cell lines used were of human origin. LS174T and HT29, adenocarcinoma of colon, SK-MEL-5, malignant melanoma and NIH:OVCAR-3 adenocarcinoma of ovary were all originally obtained from ATCC. The JAM cell line, a serous cystadenocarcinoma of ovary (Ward et al, 1987), was kindly provided by Dr Peter Parsons, Queensland Institute of Medical Research.

The cell lines were maintained in 75-$cm^2$ tissue culture flasks (Costar, USA) in RPMI 1640 (Life Technologies, USA) supplemented with 10% fetal calf serum (FCS) and 100 U $ml^{-1}$ penicillin (CSL, Australia) and for NIH:OVCAR-3 10% extra FCS and human insulin (Actrapid, Novo Nordisk, Denmark) at 10 ug $ml^{-1}$ were added to the media. Confluent cells were harvested using trypsin-versene (CSL, Australia) counted then washed twice with phosphate-buffered saline (PBS) to remove FCS and resuspended at a concentration of $10^8$ cells $ml^{-1}$ immediately before injection into sheep.

### Cell injections

Injections were given via a 21G needle as 0.1–0.3 ml of cells in PBS with or without 0.1 ml of Matrigel (Collaborative Biomedical Products, USA).

#### *Skin sites*

Approximately $10^7$ SK MEL, LS174T, HT 29 and JAM cells in PBS were injected subcutaneously 5 cm apart on the shaved sides and flanks of 13 sheep at four injection sites for each cell line.

© Cancer Research Campaign 1998 

**Figure 1** (**A**) Well-formed acinar structures in colon adenocarcinoma arising from subcutaneous injection of LS174T cells H & E stain, 400×. (**B**) Marked positive cytoplasmic staining with luminal border accentuation of LS 174T adenocarcinoma with CEA immunoperoxidase stain, 400×. (**C**) Abundant eosinophilic intraluminal mucin production by LS 174T adenocarcinoma. PAS stain post diastase, 400×. (**D**) Whole mount sections of intestinal wall with submucosal tumour deposit of LS 174T adenocarcinoma of colon. H & E stain. (**E**) Metastatic tumour deposit of LS 174T adenocarcinoma within the periportal region of the liver. H & E stain, 100×. (**F**) Macroscopic specimen of lower rectum and anus showing submucosal tumour deposits of LS 174T adenocarcinoma elevating the overlying intact rectal mucosa

 © Cancer Research Campaign 1998

*Intra-abdominal injections*

The following procedures were carried out aseptically under halothane general anaesthetic in the animal operating theatre. At laparatomy, the cells were injected with or without matrigel using a 1-ml tuberculin syringe and a 21G needle. Non-absorbing sutures were placed 2 cm from injection sites for subsequent location at laparoscopy or autopsy. The abdominal incision was closed in layers and the sheep monitored in the recovery room until post-operative recovery was complete.

***Ovarian and peritoneal wall injections*** Three sheep received $2 \times 10^7$ NIH: OVCAR-3 cells + 0.1 ml of matrigel in one ovary and two peritoneal wall sites and $2 \times 10^7$ JAM cells + 0.1 ml of matrigel in the opposite ovary and two peritoneal wall sites. Before closure of the peritoneum NIH: OVCAR-3 cells were injected into the peritoneal cavity of one sheep. In additional sheep, JAM cells were inoculated into each ovary and at subcutaneous sites.

***Colon, liver and peritoneal wall injection*** Eleven sheep received $10^7$ LS174T cells in 0.1 ml of matrigel injected into four sites along the colon or stomach wall and two sites in the liver and peritoneal wall, as well as one injection each in the liver and peritoneal wall of $10^7$ LS174T cells without matrigel. In four other sheep, LS174T cells in matrigel were inoculated at four sites in rectal submucosa, without laparotomy, by simply prolapsing the rectum with Allis forceps under halothane anaethesia.

### Monitoring tumour growth

Skin tumours were measured weekly in two dimensions with callipers and the volume calculated using the formula $(ab^2)/2$: a, representing the longest dimension and b, the shortest. Volumes are stated as mean ± s.d., with $n$ = number of tumours measured. Tumours were excised from the skin at varying times and fixed in formalin for subsequent histological examination. At autopsy, 3–6 weeks after tumour cell inoculation appropriate organs and draining lymph nodes were removed, examined macroscopically and fixed in formalin for histology. Portions of unfixed draining lymph nodes were collected in tissue culture medium, minced with scissors and pushed through a cell dissociation sieve (Sigma USA). The resultant cell suspension was washed twice and seeded into cell culture flasks with RPMI 1640 plus 20% FCS for growth of adherent cells.

### Histology and immunocytochemistry

Tissues for histopathological examination were fixed in neutral-buffered formalin and processed in the routine fashion through alcohol and xylene to paraffin. Sections were cut at 4 microns and stained with Harris haematoxylin and aqueous eosin (H and E stain).

Mucins in tissues were demonstrated using the periodic acid–Schiff reaction (PAS). Mucins were oxidized by periodate to expose aldehydes, which were demonstrated with Schiffs reagent. Any glycogen in the tissue was removed by prior treatment with fresh malt diastase. The neutral mucin appeared as bright eosinophilic amorphous material.

Carcinoembyronic antigen (CEA) was demonstrated using rabbit anti-human CEA (Dakopatts, Glostrup, Denmark) on formalin-fixed, paraffin-embedded tissue. Antigen demonstration was achieved by a peroxidase-conjugated streptavidin staining procedure. The primary antibody was first applied to the tissue sections, which were then further labelled with a biotinylated link antibody followed by a streptavidin peroxidase enzyme conjugate. The bound peroxidase enzyme was then visualized with a diaminobenzidine substrate.

### Antibody: labelling, injection and biodistribution

A5B7 anti-CEA IgG1 monoclonal antibody kindly donated by Celltech, UK, was labelled with I-131 using the Chloramine-T method (Pedley et al, 1987). Labelling efficiency was 98% without loss of immunoreactivity.

For each sheep, 0.1 mg of A5B7 antibody was labelled with 15-MBq $^{131}$I and 0.1 mg kg$^{-1}$ cold A5B7 antibody was added immediately before intravenous administration for tissue distribution studies. For tumour imaging, 185-MBq $^{131}$I was used with the same proportion of A5B7 antibodies.

Sheep were euthanased using a lethal injection of sodium pentobarbitone at 1, 3, 5 or 7 days after administration of radiolabelled antibody. The human tumour xenografts were removed and weighed, and samples of blood, liver, spleen, kidney, thyroid, heart, lung, lymph node, bone marrow and bile samples were weighed and counted for 10 min in a gamma counter (Wallac 1480 Wizard, Wallac Oy, Turku, Finland). The % DI g$^{-1}$ was calculated for each sample taken.

Sheep, under halothane anaesthesia, were imaged at 3, 5 or 7 days after intravenous [$^{131}$I]-A5B7-radiolabelled monoclonal anti-CEA antibody, using an Elscint Apex 409 gamma camera and high-energy collimator. After lethal injection of pentobarbitone, a flap of tumour-bearing skin was excised and pinned out on foam board and imaged for 20 min. Cobalt-57 markers were then placed on the tumours and scanned to mark the exact location of each tumour on the scintigraphic images.

### RESULTS

### Human LS174T and HT 29 adenocarcinoma of colon

Subcutaneous flank inoculation of LS174T and HT 29 cells gave rise to tumours that grew steadily up to day 21 but then apparently stabilized at a mean tumour volume of 1805 ± 1184 mm$^3$ ($n$ = 20) for LS174T and 931 ± 991 mm$^3$ ($n$ = 20) for HT29.

Neither of the colonic adenocarcinomas metastasized to regional lymph nodes after subcutaneous inoculation, and serum CEA levels did not rise.

On histological examination the tumour deposits of LS174T exhibited the features of a well-differentiated adenocarcinoma with prominent well-formed acinar formations throughout the tumour, lined by columnar cells with mild to moderate nuclear pleomorphism (Figure 1A). Atypical mitotic figures were present but not numerous. There was marked cytoplasmic positivity with luminal accentuation with the immunoperoxidase preparation for CEA (Figure 1B). The LS174T tumour produced abundant neutral mucin, showing marked positivity with the PAS preparation post diastase (Figure 1C).

The less well-differentiated HT29 adenocarcinoma, in contrast to the LS174T tumour deposits, showed little tendency toward acinar formation, with moderate to marked nuclear pleomorphism, including tumour giant cells. Cytoplasmic vacuolization was

© Cancer Research Campaign 1998

**Figure 2** (**A**) Subcutaneous SK MEL melanoma in diffuse sheets with no necrosis or tumour-infiltrating lymphocytes. H & E stain, 400×. (**B**) Whole mount section of a regional lymph node showing extensive deposits of metastatic SK MEL melanoma. H & E stain. (**C**) Macroscopic specimen of JAM ovarian carcinoma diffusely infiltrating both ovaries with metastatic spread into paratubal mesentry. (**D**) Paratubal lymphatic vascular permeation from ovarian tumour deposit of JAM ovarian adenocarcinoma. H & E stain, 250×. (**E**) JAM ovarian adenocarcinoma forming poorly differentiated glands adjacent to an ovarian follicle (top). H & E stain, 400×.

© Cancer Research Campaign 1998

**Figure 3** Excised skin flap from sheep flank (left panel), reversed to show human tumour xenografts of HT 29 (top) and LS 174T (second row), SK MEL (third row) and LS 174T tumours implanted directly from nude mice (bottom). Administration of iodine-131-radiolabelled A5B7 anti-CEA monoclonal antibody 72 h before ex vivo gamma imaging demonstrated discrete uptake in LS 174T tumours with minimal activity in HT 29 tumour and no activity in SK MEL melanoma xenografts (middle panel). Matching of tumour uptake of activity on the gamma camera images was performed by imaging of cobalt-57 markers shown in situ in the corresponding positions (right panel)

apparent, including occasional intracytoplasmic pseudolumina and numerous atypical mitoses were present. Only a small amount of neutral mucin was demonstrated in HT29 tumours, present as scattered intracytoplasmic mucinous globules. The immunoperoxidase preparation for CEA decorated the tumour cells in a variably weak focally accentuated cytoplasmic pattern.

Intra-abdominal tumour deposits of LS174T in peritoneum and intestinal wall (Figure 1D) displayed the characteristic histological features described above. Although some peritumoral fibrosis was present, no necrosis or tumour-infiltrating lymphocytes were seen. Metastatic LS174T tumours measuring 2 mm in diameter in liver were centred on portal tracts, suggesting tumour seeding via the portal vein (Figure 1E). The tumours were evident macroscopically, for example in lower rectum (Figure 1F).

### Human SK-MEL melanoma

Tumours grew steadily at sites of subcutaneous inoculation and attained a mean volume of 3443 ± 2734 $mm^3$ ($n = 20$) within 37 days of inoculation of $10^7$ cells in five sheep. At this time, SK-MEL cells were recovered from the pre-stifle lymph node that drains the flank bearing the subcutaneous melanoma xenografts. Cells grown in culture from these regional lymph nodes then gave rise to SK-MEL tumours when subsequently reinoculated into other immunosuppressed sheep. The growth curve of reinoculated cells shifted slightly to the left, but the histological appearance remained consistent.

Tumour deposits of SK-MEL were present in diffuse sheets of large pleomorphic polygonal and cuboidal cells with abundant amphophilic cytoplasm, enlarged vesicular nuclei and prominent eosinophilic nucleoli. Atypical mitoses were easily identified. No significant necrosis was evident, and there was only a variable mild inflammatory host response with infrequent tumour-infiltrating lymphocytes (Figure 2A). The tumour cells showed markedly positive staining with S100 protein.

Spontaneous metastasis to draining regional lymph nodes was observed (Figure 2B), and the metastatic nodal deposits showed similar architectural and cytological features to those of the primary tumour deposit (Figure 2A).

### Human JAM and NIH:OVCAR-3 cystadenocarcinoma of ovary

JAM cells inoculated subcutaneously in the flank gave rise to tumours attaining a mean volume 653 ± 376 $mm^3$ by day 28. If co-injected with Matrigel, subcutaneous JAM cell tumours attained a volume of 1026 ± 1043 $mm^3$ within the same period. Tumour cell spread to regional lymph nodes from subcutaneous inoculation sites of JAM cells was demonstrated by histology and recovery of JAM cells by tissue culture of the minced pre-stifle lymph nodes.

Subcutaneous inoculation of NIH:OVCAR-3 cells did not give rise to tumours in the 28-day period of observation.

Inoculation of JAM cells in Matrigel directly into the ovary gave rise to tumours within 21 days in all animals.

Bilateral ovarian tumour deposits and extensive paratubal masses (Figure 2C) were typical at 1 month after direct ovarian inoculation of JAM cells. Histopathological examination confirmed paratubal lymphatic permeation (Figure 2D). There were predominantly diffuse sheet-like arrangements of pleomorphic malignant cells showing variable cytological features. Tumour giant cells were prominent with moderate to marked nuclear pleomorphism, and atypical mitoses were easily identified (Figure 2E). No definite papillary structures were present. Subperitoneal inoculation of JAM cells in Matrigel was also productive of tumours at all sites with similar histological appearance.

Administration of NIH:OVCAR-3 tumour cells directly into the peritoneal cavity resulted in bladder tumour deposits exhibiting a predominantly diffuse architecture with occasional slit-like spaces. The neoplastic cells exhibited variable cytological features with cuboidal and spindeloid cells admixed with markedly pleomorphic and occasionally multinucleated cells. Marked nuclear pleomorphism was evident and atypical mitoses easily identified. A mild chronic inflammatory host response was present, but there

© Cancer Research Campaign 1998

was no tumour necrosis and only small numbers of tumour-infiltrating lymphocytes were evident focally, confined to the periphery of the tumour deposits.

### Imaging and uptake

Subcutaneous LS174T and HT29 tumour xenografts could not be defined in vivo by gamma camera imaging because of relatively high blood background activity. However, ex vivo imaging of the tumour-bearing skin flap did demonstrate foci of tumour-specific uptake in LS174T, and to a lesser extent in xenografts of HT29 (Figure 3). There was no visible activity in the control SK MEL tumours. The mean uptake of [$^{131}$I]A5B7 in LS174T colon cancer xenografts and normal tissues in the immunosuppressed sheep (Table 1) demonstrated highest tumour to blood ratios of 2–2.5 at 5 days post injection of antibody when mean uptakes of 0.034% DI $g^{-1}$ in liver metastases and 0.027% DI $g^{-1}$ in intestinal LS174T xenografts were achieved. At each time point, uptake of [$^{131}$I]A5B7 was highest in LS174T xenografts, next highest in HT29 tumours, which was greater than normal liver activity and uptake was least in SK-MEL tumours.

### DISCUSSION

The great expectations of radioimmunotherapy of cancer metastasis raised by encouraging results in human tumour xenografts treated in nude mice have yet to be realized in patients (Sgouros, 1995).

Extrapolation from nude mouse xenograft tumour models to humans is primarily limited by the relatively small size and volume of distribution in mice that promotes uptake of systemically administered antibody in transplanted human tumour to levels unattainable in patients (Knox, 1995). There are also significant differences between humans and mice in self-absorption of radiation, bone marrow radiosensitivity and repopulation kinetics, tumour cell cycle time and volume-doubling time and the presence of cross-reactive antigens (Wahl, 1994). To avoid these problems, we have developed a large animal model of human tumours in immunosuppressed sheep. We have previously shown that cyclosporin effectively abrogates immunocompetence in sheep and that this immunosuppressive effect is enhanced by concomitant administration of ketoconazole (O'Donoghue et al, 1996), just as is observed in man (Schroeder et al, 1987). We have also shown that daily combined therapy comprising intravenous cyclosporin via in-dwelling jugular vein catheter and oral ketoconazole given in a drench formulation to bypass the rumen and facilitate abomasal absorption will effectively suppress the immune response for several weeks. Biochemical monitoring of these animals showed no evidence of nephrotoxicity or hepatotoxicity if trough blood levels of cyclosporin remained below 1500 ng $ml^{-1}$, above which the sheep became anorexic and lost condition. The apparent tolerance of sheep to maintenance of cyclosporin at these relatively high blood levels contrasts with the renal transplant experience in humans, in whom trough levels of 500 ng $ml^{-1}$ immediately after kidney grafting have to be reduced to maintenance levels of typically 100–200 ng $ml^{-1}$ to avoid cyclosporin-induced nephrotoxicity.

We have previously observed that our chronically immunosuppressed sheep tolerate full-thickness heterologous skin grafts without histological evidence of rejection, provided that trough blood cyclosporin levels are maintained above 750 ng $ml^{-1}$. At the optimum serum level of 1000 ng $ml^{-1}$, the sheep remained healthy with no apparent increase in susceptibility to infection. They had no requirement for prophylatic antibiotics, sterile feed or aseptic environment. Sheep were penned together and aseptic conditions prevailed only during surgical procedures. Thus, the cyclosporin-immunosuppressed sheep offers a practical and relatively robust model that can be used to study human tumours in an animal of comparable size.

Adequacy of immunosuppression of the sheep was reflected in the minimal histological features of regression in all the human tumour transplants examined. Most tumour deposits showed a mild lymphocyte inflammatory host response without significant necrosis and no tumour-infiltrating lymphocytes. Some deposits were surrounded by a marked chronic inflammatory host response with or without a desmoplastic mesenchymal proliferation, and this tended to occur in exposed sites of inoculation, such as skin and gastrointestinal submucosal deposits, perhaps in response to an additional antigenic stimulation.

Subcutaneous inoculation of human tumour cells in the flanks of sheep may be performed easily at multiple sites without anaesthesia. Given optimum cyclosporin levels, virtually all inoculates of $10^7$ cells of LS174T and HT29 human colon carcinoma and SKMEL human melanoma and JAM human ovarian carcinoma grew to a size of 1–2 cm over a period of 3 weeks. In general, the individual tumour cell morphology in our animals remained unchanged but some tumour deposits showed a variable degree of dedifferentiation. For instance, LS174T deposits often exhibited focal areas of the classical well-differentiated large acinar formations merging with poorly differentiated areas, and some deposits were entirely poorly differentiated. The practical significance of these findings, particularly in reference to CEA expression, potential for aggressive tumour behaviour and tumour uptake of anti-CEA monoclonal antibody have yet to be determined in this animal model. Histological examination did, however, demonstrate maintenance of the comparative dedifferentiation of HT29 tumour cells in comparison with LS174T, in which the well defined acinar structures were shown to produce more mucin and had greater CEA expression on immunoperoxidase staining. These differences were reflected in the relatively greater tumour uptake of anti-CEA monoclonal antibody in LS174T than in HT29 human colon cancer xenografts. Weekly monitoring of serum CEA levels in sheep bearing colon cancer xenografts did not show any elevation, which accords with data from LS174T xenografts in nude mice (Pedley et al, 1987). The SKMEL tumours showed typical morphological characteristics of human melanoma and did not accumulate radiolabelled anti-CEA antibody.

Evidence of some loss of differentiation was observed in both colon and ovarian carcinomas. Dedifferentiation was focal in the LS174T tumour deposits but was evident throughout the xenografts of JAM and NIH: OVCAR-3 ovarian serous adenocarcinoma, in which the expected papillary formations were not observed. It is possible that this relative tumour dedifferentiation may have been the result of multiple passaging of cell lines, but further study is required to exclude various host factors.

The melanoma was observed to metastasize from subcutaneous sites of inoculation, and SKMEL cells were subsequently recovered from the regional pre-stifle lymph node, grown in cell culture and reinoculated subcutaneously in sheep, giving rise to tumours morphologically indistinguishable from those of the primary inoculation. JAM ovarian carcinoma also metastasized to regional

© Cancer Research Campaign 1998

lymph nodes draining both subcutaneous and ovarian sites of inoculation. The failure of tumours to metastasize from subcutaneous sites in nude mice models is well documented (Fidler, 1990; Kubota, 1994), and Manzotti et al (1993) have reviewed the importance of orthotopic transplantation of human tumours in relation to metastasis and invasion.

Orthotopic transplantation of LS174T human colon cancer in the sheep was achieved by inoculation of $10^7$ cells into the wall of stomach and colon, and hepatic metastases were induced by intravenous administration by portal vein or simulated by intrahepatic inoculation. Direct subperitoneal implantation was also successful. Spontaneous metastasis to liver or lymph nodes was not observed in these animals possibly because of the relatively short duration of the experiment (3 weeks) and not having a reliable technique, as yet, for locating small numbers of tumour cells deposited within the liver and lymph nodes. Studies of metastasis may be facilitated by orthotopic implantation of intact human tumours (Fu et al, 1991), which would be relatively easy in sheep in comparison with mice and may be performed at multiple sites in the same animal. In later experiments, we achieved orthotopic xenografts of LS174T colonic carcinoma without laparatomy, simply by inoculating $10^7$ cells in Matrigel submucosally in the partly everted rectum, under halothane anaesthesia. Monitoring of these tumours by digital rectal examination was simple and serial biopsy can be performed under direct vision via proctoscopy. This location more accurately reflects the location of the tumours in humans compared with stomach and small intestine and, with further experiments of longer duration, may give rise to metastasis.

Matrigel, a reconstituted basement membrane matrix (Fridman et al, 1991), was found to facilitate tumour take at sites of cell inoculation particularly for NIH: OVCAR-3 and JAM human ovarian carcinoma cells orthotopically transplanted into sheep ovaries. Enhancement of tumour growth was also observed after transplantation of multicell spheroids of LS174T cells in comparison with inoculation of LS174T single-cell suspension at the same sites. We also found that LS174T xenografts grown subcutaneously in nude mice from cell inoculations, when implanted into the immunosuppressed sheep subdermally grew more rapidly than xenografts arising from inoculation of LS174T single-cell suspensions. The uptake of [$^{131}$I]A5B7 anti-CEA monoclonal antibody was similar for such implanted tumour chunks to that observed in subcutaneous LS174T xenografts originating from inoculation of cell suspensions, and both were demonstrated on ex-vivo gamma camera images taken 3–5 days after administration of the radiolabelled anti-CEA antibody (Figure 3).

The tumour uptake in LS174T xenografts shown in Table 1 is in accord with that achieved in human colonic tumour studies in patients using [$^{131}$I]A5B7 anti-CEA monoclonal antibody, when peak uptake of 0.018% DI $g^{-1}$ was observed at 27 h after administration of radiolabelled intact antibody (Lane et al, 1994). These modest tumour uptakes in sheep contrast with those achieved in LS174T human colon cancer xenografts in nude mice in which [$^{131}$I]A5B7 peak tumour uptake is over 20% DI $g^{-1}$ (Pedley et al, 1993). Not only is tumour uptake of antibody much higher in LS174T xenografts in nude mice, but comparison of the distribution of the same radiolabelled antibody in a mouse model and in patients has shown different distribution in tumour and normal tissues (Begent et al, 1990). In contrast, we have shown that the distribution of [$^{131}$I]A5B7 anti-CEA monoclonal antibody between human LS174T colon cancer xenografts in sheep and normal ovine tissues (Table 1) is comparable with that reported in patients with colon carcinoma using $^{131}$I-anti-CEA-radiolabelled antibody (Begent et al, 1989), although blood clearance was slower.

Relatively high human tumour uptakes of radiolabelled antibodies are commonly achieved in nude mouse xenografts (Senekowitsch et al, 1989; Siler et al, 1993), but the typical uptakes for the same antibody and tumour type in man are around 0.005% DI $g^{-1}$ (Dykes et al, 1987; Begent et al, 1990). Expectations of curability of tumours by radioimmunotherapy based on nude mouse results are therefore unrealistic. For example, if a 60-Gy dose in 1 week is considered sufficient for tumour sterilization, and given a tumour uptake of $^{131}$I-labelled monoclonal antibody of 0.005% DI $g^{-1}$, the corresponding whole-body radiation absorbed dose would be 17 Gy (Vaughan et al, 1986). The maximum tolerable whole-body dose in man is in fact around 2 Gy and new approaches to radioimmunotherapy of solid tumours will be necessary. One such approach is regional therapy, and the comparable size and anatomy of the sheep will facilitate exploration of methods of local and intratumoral radioimmunotherapy. For example, we have inoculated our immunosuppressed sheep with human tumour cells in liver and in peritoneum to provide models for regional radioimmunotherapy delivered via hepatic artery or intra-peritoneal injection. Monitoring by quantitative gamma camera imaging is easily performed in this large animal model. In addition, results can be correlated with counting of serial biopsy samples and autoradiography to validate algorithms for calculation of dosimetry in patients in subsequent clinical trials to evaluate safety and efficacy of radioimmunotherapy of cancer.

Modelling of micrometastasis in the immunosuppressed sheep may be contemplated, for instance, by surgical removal of the 'primary' site of ovarian xenografting of JAM cell tumour after metastasis has occurred. Subsequent treatment with intraperitoneal radiolabelled monoclonal antibodies and second-look surgery may allow more complete evaluation of the efficacy of adjuvant radioimmunotherapy than can be performed in clinical trials. Other modalities of adjuvant therapy may conceivably be tested in the sheep model. For example, the efficacy and toxicity of external-beam radiotherapy for adjuvant treatment of rectal carcinoma may potentially be assessed in ovine rectal tumour xenografts of human LS174T colon cancer.

We have demonstrated that a variety of human cancers may be orthotopically xenografted in immunosuppressed sheep. This large animal model has the potential for preclinical evaluation of novel treatments of human tumours under controlled experimental conditions that approach the human condition much more closely than those prevailing in a nude mouse. Assessment of adjuvant treatments, such as radioimmunotherapy, of human tumour xenografts in immunosuppressed sheep may thus improve prediction of their therapeutic efficacy in patients.

### ACKNOWLEDGEMENTS

We wish to thank Andrew Martindale and Magda Carcione for nuclear medicine technology assistance, Denham Heliams and Megan Carter for animal welfare, Linda Manning and Helen O'Donoghue for performing preliminary experimental studies and Ken Ilett for pharmacological advice and provision of cyclosporin assays. We also gratefully acknowledge Paul Norman, Cameron Platell and Andrew Barker who performed intra-abdominal surgery, Len Maker and Peter Hall who prepared the pathology specimens and photographs, Greg Sheridan for haematology and Jenny Lavin for processing the manuscript.

© Cancer Research Campaign 1998

The cyclosporin was kindly donated by Sandoz Pharma (Basle) (now Novartis), and the encouragement and support of Jurg Schaedlin and William Harrison is acknowledged with thanks. Celltech (Slough, UK) generously donated A5B7 antibody, and the expert advice from David King in respect of radiolabelling was much appreciated. Degussa Australia, for donating Ultrasil VN3 for the drench formulation, subsidy of ketoconazole by Janssen-Cilag and of EMIT assays by Syva Co are also gratefully acknowledged. The study was funded by a National Health and Medical Research Council Project Grant.

### REFERENCES

Begent RHJ and Pedley RB (1990) Antibody targeted therapy in cancer: comparison of murine and clinical studies. *Cancer Treat Rev* **17**: 373–378

Begent RHJ, Ledermann JA, Green AJ, Bagshawe KD, Riggs SJ, Searle F, Keep PA, Adam T, Dale RG and Glaser MG (1989) Antibody distribution and dosimetry in patients receiving radiolabelled antibody therapy for colorectal cancer. *Br J Cancer* **60**: 406–412

Blumenthal RD, Sharkey RM, Haywood L, Natale AM, Wong GY, Siegel JA, Kennel SJ and Goldenberg DM (1992) Targeted therapy of athymic mice bearing GW39 human colonic cancer micrometastases with $^{131}$I-labelled monoclonal antibodies. *Cancer Res* **52**: 6036–6044

DeNardo GL, Kroger LA, DeNardo SJ, Miers LA, Salako Q, Kukis DL, Fand I, Shen S, Renn O and Meares CF (1994) Comparative toxicity studies of Yttrium-90 MX-DTPA and 2-IT-BAD conjugated monoclonal antibody (BrE-3). *Cancer* **73** (suppl.): 1012–1022

Dykes PW, Bradwell AR, Chapman CE and Vaughan ATM (1987) Radioimmunotherapy of cancer: clinical studies and limiting factors. *Cancer Treat Rev* **14**: 87–106

Fidler I (1990) Critical factors in the biology of human cancer metastasis: Twenty-eighth G.H.A. Clowes Memorial Award Lecture. *Cancer Res* **50**: 6130–6138

Fridman R, Kibbey MC, Royce LS, Thomas MZ, Sweeney M, Jicha DL, Yanelli JR, Martin GR and Kleinman HK (1991) Enhanced tumour growth of both primary and established human and murine tumour cells in athymic mice after coinjection with matrigel. *J Natl Cancer Inst* **83**: 769–774

Fu X, Besterman JM, Monosov A and Hoffman RM (1991) Models of human metastatic colon cancer in nude mice orthotopically constructed by using histologically intact patient specimens. *Proc Natl Acad Sci USA* **88**: 9345–9349

Kallinowski F, Schlenger KH, Runkel S, Kloes M, Stohrer M, Okunieff P and Vaupel P (1989) Blood flow metabolism, cellular microenvironment and growth rate of human tumour xenografts. *Cancer Res* **49**: 3759–3764

Knox SJ (1995) Overview of studies on experimental radioimmunotherapy. *Cancer Res* **55** (suppl.): 5832–5836

Kubota T (1994) Metastatic models of human cancer xenografted in the nude mouse: the importance of orthotopic transplantation. *J Cell Biochem* **56**: 4–8

Lane DM, Eagle KF, Begent RHJ, Hope-Stone LD, Green AJ, Casey JL, Keep PA, Kelly AMB, Glaser MG and Hilson AJW (1994) Radioimmunotherapy of metastatic colorectal tumours with iodine-131-labelled antibody to carcinoembryonic antigen: phase I/II study with comparative biodistribution of intact and F(ab')$_2$ antibodies. *Br J Cancer* **70**: 521–525

Ledermann JA, Begent RHJ, Massof C, Kelly AMB, Adam T and Bagshawe KD (1991) A phase-1 study of repeated therapy with radiolabelled antibody to carcinoembryonic antigen using intermittent or continuous administration of cyclosporin A to suppress the immune response. *Int J Cancer* **47**: 659–664

Manzotti C, Riccardo A and Pratesi G (1993) Importance of orthotopic implantation for human tumors as model systems: relevance to metastasis and invasion. *Clin Exp Metastasis* **11**: 5–14

O'Donoghue HL, Penhale WJ, Manning LS, Reynoldson JA and Turner JH (1996) Cyclosporine A immunosuppression in sheep with response enhancement by concomitant ketoconazole. *Clin Exp Pharmacol Physiol* **23**: 797–803

Pedley RB, Boden J, Keep PA, Harwood PJ, Green AJ and Rogers GT (1987) Relationship between tumour size and uptake of radiolabelled anti-CEA in a colon tumour xenograft. *Eur J Nucl Med* **13**: 197–202

Pedley RB, Boden JA, Boden R, Dale R and Begent RHJ (1993) Comparative radioimmunotherapy using intact of F(ab')$_2$ fragments of $^{131}$I anti-CEA antibody in a colonic xenograft model. *Br J Cancer* **68**: 69–73

Rygaard J and Povlsen CO (1969) Heterotransplantation of a human malignant tumour to nude mice. *Acta Pathol Microbiol Scand* **77**: 758–760

Schroeder TJ, Melvin DB, Clardy CW, Wadhwa NK, Myre SA, Reising JM, Wolf RK, Collins JA, Pesce AJ and First MR (1987) Use of cyclosporine and ketoconazole without nephrotoxicity in two heart transplant recipients. *J Heart Transplant* **6**: 84–89

Senekowitsch R, Reidel G, Mollenstadt S, Kriegal H and Pabst HW (1989) Curative radioimmunotherapy of human mammary carcinoma xenografts with Iodine 131-labeled monoclonal antibodies. *J Nucl Med* **30**: 531–537

Sgouros G (1995) Radioimmunotherapy of micrometastases: side-stepping the solid-tumour hurdle. *J Nucl Med* **36**: 1910–1912

Siler K, Eggensperger D, Hand PH, Milenic DE, Miller LS, Houchens DP, Hinkle G and Schlom J (1993) Therapeutic efficacy of a high-affinity anticarcinoembryonic antigen monoclonal antibody (COL-1). *Biotech Ther* **4**: 163–181

Vaughan ATM, Bradwell AR, Dykes PW and Anderson P (1986) Illusions of tumour killing using radiolabeled antibodies. *Lancet* **1**: 1492–1493

Wahl RL (1994) Experimental radioimmunotherapy. *Cancer* **73** (suppl.): 989–992

Ward BG, Wallace K, Shepherd JH and Balkwill FR (1987) Intraperitoneal xenografts of human epithelial ovarian cancer in nude mice. *Cancer Res* **47**: 2662–2667

Yu B, Carrasquillo J, Milenic D, Chung Y, Perentesis P, Feuerestein I, Eggensperger D, Qi C-F, Paik C, Reynold J, Grem J, Curt G, Siler K, Schlom J and Allegra C (1996) Phase 1 trial of Iodine 131-labeled COL-1 in patients with gastrointestinal malignancies: influence of serum carcinoembryonic antigen and tumour bulk on pharmacokinetics. *J Clin Oncol* **14**: 1798–1809

© Cancer Research Campaign 1998